# Efficient handling of ACL policy change in SDN using reactive and proactive flow rule installation

**DOI:** 10.1038/s41598-024-65721-x

**Published:** 2024-06-28

**Authors:** Mudassar Hussain, Rashid Amin, Rahma Gantassi, Asma Hassan Alshehri, Jaroslav Frnda, Syed Mohsan Raza

**Affiliations:** 1 Department of Computer Science and Creative Technologies, Global College of Engineering and Technology, CPO Ruwi 112, P.O Box 2546, Muscat, Sultanate of Oman; 2 Department of Computer Science and IT, University of Chakwal, Chakwal, 48800 Pakistan; 3 Department of Computer Science, University of Engineering and Technology, Taxila, 47050 Pakistan; 4 Department of Electrical Engineering, Chonnam National University, Gwangju, 61186 South Korea; 5 Department of Computer Science, College of Computer Engineering and Science, Prince Sattam Bin Abdulaziz University, Alkharj, 16273 Saudi Arabia; 6 Department of Quantitative Methods and Economic Informatics, Faculty of Operation and Economics of Transport and Communications, University of Zilina, 01026 Zilina, Slovakia; 7 Department of Telecommunications, Faculty of Electrical Engineering and Computer Science, VSB Technical University of Ostrava, 70800 Ostrava, Czech Republic; 8 SAMOVAR, Telecom SudParis, Institut Polytechnique de Paris, 91120 Paris, France

**Keywords:** SDN, Security, Management, Proactive flow rule installation, Reactive flow rule installation, ACL policy change, Electrical and electronic engineering, Mathematics and computing

## Abstract

Software-defined networking (SDN) is a pioneering network paradigm that strategically decouples the control plane from the data and management planes, thereby streamlining network administration. SDN's centralized network management makes configuring access control list (ACL) policies easier, which is important as these policies frequently change due to network application needs and topology modifications. Consequently, this action may trigger modifications at the SDN controller. In response, the controller performs computational tasks to generate updated flow rules in accordance with modified ACL policies and installs flow rules at the data plane. Existing research has investigated reactive flow rules installation that changes in ACL policies result in packet violations and network inefficiencies. Network management becomes difficult due to deleting inconsistent flow rules and computing new flow rules per modified ACL policies. The proposed solution efficiently handles ACL policy change phenomena by automatically detecting ACL policy change and accordingly detecting and deleting inconsistent flow rules along with the caching at the controller and adding new flow rules at the data plane. A comprehensive analysis of both proactive and reactive mechanisms in SDN is carried out to achieve this. To facilitate the evaluation of these mechanisms, the ACL policies are modeled using a 5-tuple structure comprising Source, Destination, Protocol, Ports, and Action. The resulting policies are then translated into a policy implementation file and transmitted to the controller. Subsequently, the controller utilizes the network topology and the ACL policies to calculate the necessary flow rules and caches these flow rules in hash table in addition to installing them at the switches. The proposed solution is simulated in Mininet Emulator using a set of ACL policies, hosts, and switches. The results are presented by varying the ACL policy at different time instances, inter-packet delay and flow timeout value. The simulation results show that the reactive flow rule installation performs better than the proactive mechanism with respect to network throughput, packet violations, successful packet delivery, normalized overhead, policy change detection time and end-to-end delay. The proposed solution, designed to be directly used on SDN controllers that support the Pyretic language, provides a flexible and efficient approach for flow rule installation. The proposed mechanism can be employed to facilitate network administrators in implementing ACL policies. It may also be integrated with network monitoring and debugging tools to analyze the effectiveness of the policy change mechanism.

## Introduction

SDN, as a novel network architecture enables decoupling of the network control and management of switches/routers from the data forwarding plane^1^. This decoupling enables centralized control through an SDN controller, serving as the network's intelligence, while leaving the data plane functions within the switches. The management plane encompasses network applications like ACL policy enforcement, monitoring, and load balancing, customized by network administrators based on application requirements and user needs^2–5^. Standardized APIs are employed to facilitate communication between SDN planes, including the Southbound Interface (SBI) for data plane-control plane interaction, predominantly utilizing the secure OpenFlow Protocol (OFP). The Northbound Interface (NBI) also facilitates control plane-management plane communication, enabling seamless coordination and implementation of network policies^6^. The SDN architecture's programmability and flexibility, supported by SBI and NBI interfaces, enhance network manageability, control, and scalability, revolutionizing the traditional networking paradigm. Data communication in networks relies on ACL policies, load-balancing applications, etc. ACL policies comprise a collection of flow rules that control network traffic flow by determining what traffic is allowed or blocked by a network device^7^.

The policies are configured based on source IP address, destination IP address, port numbers, protocols, and other attributes. These policies play a crucial role in network security by regulating traffic flow and protecting resources from unauthorized access^8–11^. The SDN programming languages facilitate stating these policies by using parallel and sequential composition operators. SDN controller translates these policies and generates flow rules which are installed at switches. The translation of conflict-free ACL policies and installation of flow rules at the switches is still a challenge that needs serious attention. Additionally, in communication networks, due to the dynamic nature of the application environment, network topology, and user requirements rapid changes in ACL policies occur. Although, SDN offers the ability to centralize network management, making it easier to configure ACL policies. However, handling ACL policy change efficiently in SDN is still challenging^12^.

As SDN networks grow, the number of flow rules and the complexity of policies can become increasingly challenging to manage^13–16^. When ACL policies change, it is essential to propagate these changes quickly and accurately across the networks. However, as the number of devices and network nodes in SDN networks grows, managing these changes can become increasingly difficult and may lead to errors and inconsistencies. Similarly, consistency is also essential when managing ACL policy change in SDN. Policies not consistently applied across the network can result in security breaches, data leaks, and other network issues. Finally, the efficient handling of ACL policy change in SDN may significantly impact the network performance. In traditional networks, ACL policies are typically enforced on network devices, which can lead to performance bottlenecks. Some studies discuss the other network management techniques^17–20^. SDN enables the controller to seamlessly enforce ACL policies by deploying corresponding flow rules at the switches. However, it becomes challenging when these policies change over time, which is quite frequent in production networks. This rapid change in ACL policies leads to packet violations and network inefficiencies. It is because of previously installed flow rules as per old ACL policies which still exist in the flow tables of switches.

**Figure 1:**
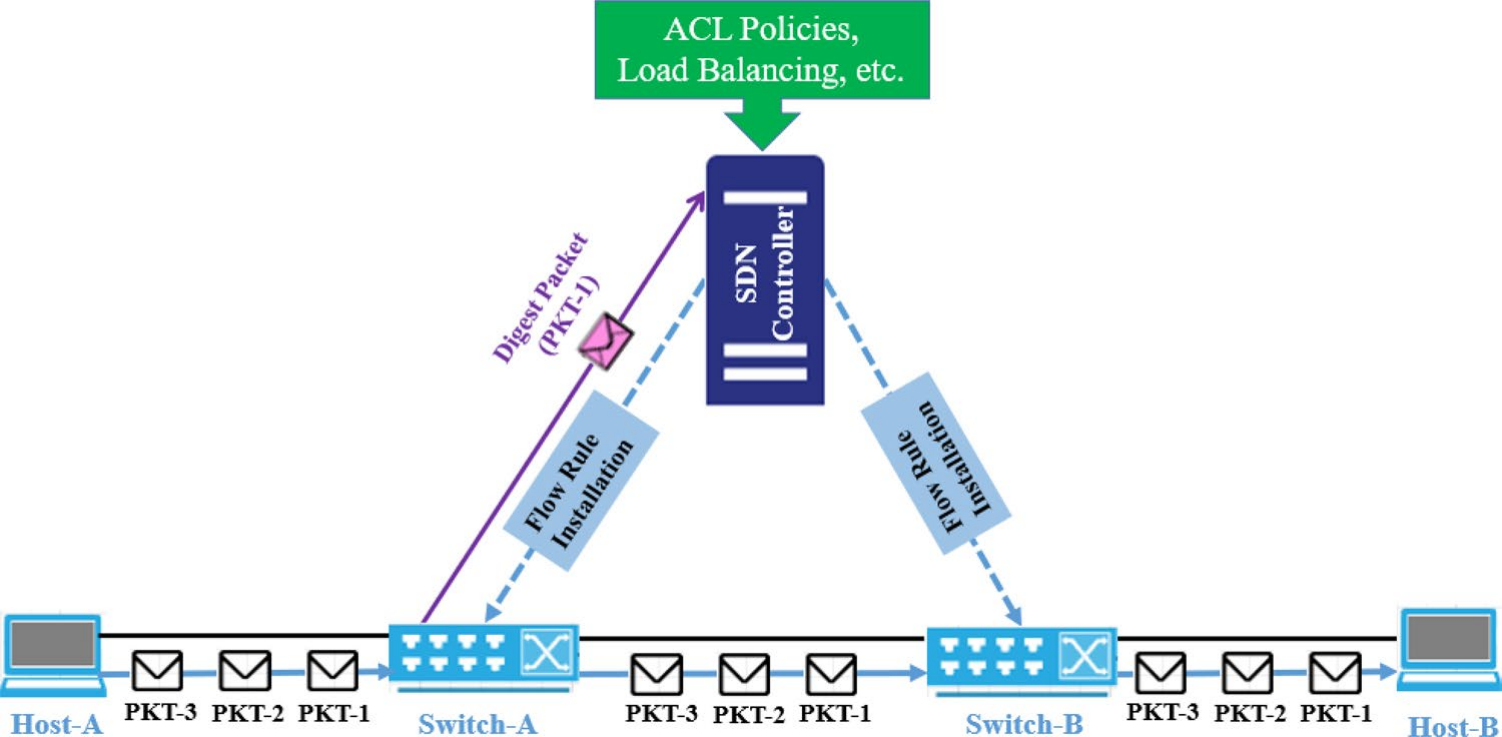
Reactive flow rule installation mechanism.

Installing flow rules in SDN can be achieved through two main mechanisms. In the reactive flow rule installation mechanism, a flow rule is installed at a switch when it receives the first flow packet for which there is no existing entry in its flow table to process the packet. To inform the controller about this new flow, a digest packet is sent to the controller using the OFP. In SDN, the OFP is a facilitator between controller and the switches. By utilizing OFP, the controller comprehensively understands the network, including its topology and ACL policies. These are used to compute the optimal path and determine the appropriate flow rules for efficient packet forwarding. This approach enables the controller to have a global network view and make informed decisions regarding flow rule installation and management. After the initial flow rule installation, the subsequent communication occurs without involving the controller, thus reducing overhead. This reactive flow rule installation process is illustrated in Fig. 1, where Host-A wants to communicate with Host-B. When the first packet (PKT-1) arrives at Switch-A, it checks its flow table and does not find a matching entry. Consequently, it sends a digest packet to the controller, requesting the installation of the corresponding flow rules along the path. Once the necessary flow rules are installed, the first data packet (PKT-1) is forwarded from Switch-A. Importantly, the flow rules remain in the flow tables of switches (Switch-A, Switch-B) to facilitate the delivery of subsequent packets (PKT-2 and PKT-3) without needing controller intervention. This mechanism effectively reduces switch flow table congestion, optimizing the utilization of Ternary Content Addressable Memory (TCAM) resources. TCAM is a high-speed memory commonly used in network devices for storing and performing fast lookups of Access Control Lists (ACLs) and other flow rules.

**Figure 2:**
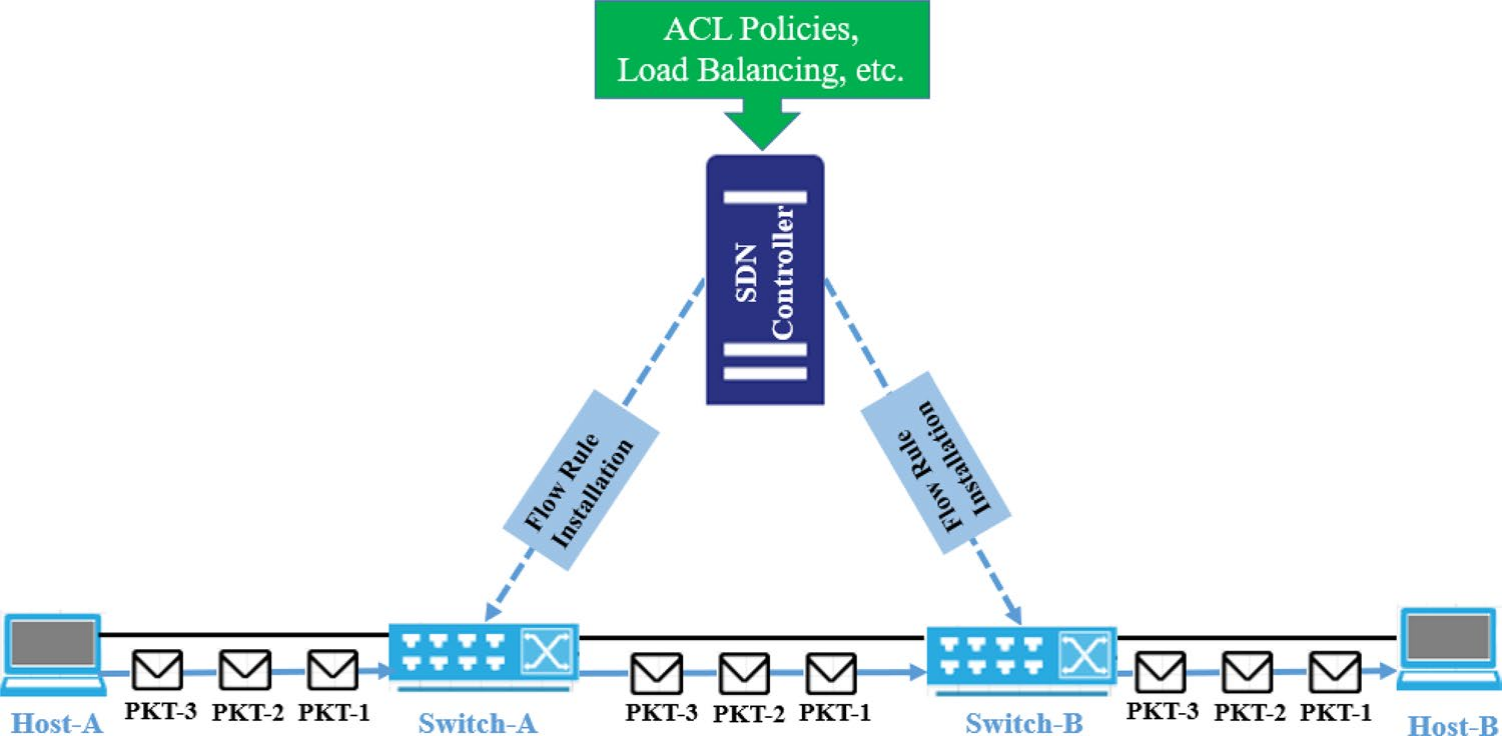
Proactive flow rule installation mechanism.

In the proactive flow rule installation mechanism, the flow rules are installed in forwarding devices before the arrival of the first packet (PKT-1) at the switch as shown in Fig. 2. It means that all packets will be forwarded as per policies and there will be the least communication of control traffic between the controller and switches to deal with flow rule provisioning in topology. The flow rule installation mechanism is based on the domain administrator's intents, including ACL policies, load balancing applications, routing protocols, etc. The proactive mechanism is efficient in decreasing the flow setup delay due to the predefined actions before the packets reach the switches. It saves time for packet delivery; however, this mechanism is inefficient as the flow rules for those hosts will also be installed where there will be no communication. Thus, it creates extra overhead in installing and storing unwanted flow rules. The flow rules installed at switches are retained in the flow tables until a timeout period elapses. These timeout values are divided into two categories: soft and hard. Soft timeout refers to the duration of inactivity, where no packets match for a specific flow rule. After this period of inactivity, the flow rule is removed from the flow table. On the other hand, hard timeout specifies a fixed time duration after which the flow rule is automatically flushed from the flow table, regardless of packet activity. This distinction between soft and hard timeouts helps to manage the flow table entries efficiently and ensure that inactive flow rules are cleared to optimize resource utilization^3^. The timeout duration for different controller platforms may vary and timeout duration could be hard-coded based on specific target scenarios^4^.

The existing studies^21,22^ have contributed to reducing packet violations and improving network throughput in scenarios where there is a change in ACL policies. However, these studies are based on the reactive flow rule installations. There is still a dire need to evaluate reactive and proactive flow rule installation. SDN implementation is gaining widespread acclaim for its exceptional management simplicity and centralized control. Google and Facebook are among the leading technology companies that have embraced SDN. These advanced data centers and campus networks necessitate frequent and dynamic updates to ACL policies, enabling precise communication regulation between hosts based on application and user-specific requirements. There is currently no research carried out that can detect ACL policy change in SDN and install flow rules proactively or reactively as per the requirements of applications/users. The reactive flow rule installation is normally desired in scenarios where traffic variability, cost-effective resource allocation, adaptive network behavior, traffic engineering, and ACL policy implementation are important factors. The proactive flow rule installation is normally desired in scenarios where traffic patterns are predictable, QoS guarantees are required, multicast or broadcast traffic is prevalent, control plane communication needs to be minimized, energy efficiency is a priority, or load balancing and traffic optimization are crucial. These factors lead us to analyze the ACL policy change mechanism and flow rule installation problem in SDN using proactive and reactive mechanisms. To accomplish this, the ACL policies are structured using a 5-tuple model consisting of < Source, Destination, Protocol, Ports, Action > . Subsequently, these policies are transformed into a policy implementation file relayed to the controller for execution. The interaction between the Source and Destination undergoes evaluation to determine whether it is permitted or denied in accordance with the ACL policy constraints.

The controller leverages the network topology and ACL policies to calculate the appropriate flow rules for the specific communication flow. These flow rules are subsequently deployed at the switches and a copy of these rules is cached at controller. In proactive scenarios all flow rules are installed before the start of communication, however, in reactive scenarios, the flow rules are installed as per demand. In the event of a policy change, the controller employs multi-attributed graphs and matrices to detect and analyze the modifications. Suppose no discrepancies are found upon comparison, indicating that the ACL policies remain unchanged. In that case, no further action is necessary as the network continues to operate in accordance with the updated ACL policies. However, if conflicting changes in ACL policies are identified, the controller determines whether they clash with the cached flow rules. Flow rules that conflict with the changes are categorized as conflicting, while those that do not conflict are classified as non-conflicting. The new policy is appended to the existing rules for the specific Source and Destination hosts for non-conflicting flow rules. Conversely, if conflicts arise, the conflicting flow rules are removed from the switches' flow tables and the controller's cache. Subsequently, fresh flow rules are computed based on the new policy and then installed at the switches along the shortest path in addition to caching at the controller.

### Research contributions

Our research makes several significant contributions in addressing a crucial network challenge related to ACL policy change in SDN environments and conducting a comprehensive analysis of the problem concerning reactive and proactive installation of flow rules. To the best of our knowledge, our research addresses a significant network challenge pertaining to ACL policy change in SDN environments. It provides a rigorous analysis of the problem in relation to reactive and proactive installation of flow rules. Our work aims to empower network administrators in effectively specifying, implementing, debugging, and managing ACL policies, while also aiding vendors in designing forwarding devices with advanced features for seamless ACL policy implementation. To tackle this research problem, we employed the pyretic language to model ACL policies at a high-level, abstract view, surpassing the limitations of low-level commands. Leveraging both Matrix-Based (Efficient Policy Enforcement—EPE)^21^ and Graph-Based (Graph-Based Policy Enforcement—GPE)^22^ approaches, we successfully detected and implemented ACL policy changes at the controller level. Upon policy change detection, we computed the necessary flow rules and installed them reactively or proactively at the switches based on application or user requirements, simultaneously caching them at the controller using a hash table data structure. In this process, we utilized a Consistent Hash Function to ensure accurate retrieval of specific flow rules on designated switches. The computed flow rules were then installed by the controller along the shortest path from the controller, effectively replacing conflicting old flow rules between the source and destination.

The simulation results indicate a significant difference in packet violation percentages between the proactive and reactive mechanisms when dealing with ACL policy changes. The proactive approach demonstrates higher packet violations compared to the reactive mechanism. Furthermore, the reactive flow rule installation performs better regarding successful packet delivery percentage and network throughput. On the other hand, the proactive installation incurs a higher normalized overhead ratio due to the increased computational overhead associated with policy changes. These findings highlight the trade-off between proactive and reactive approaches, with the reactive method showing better outcomes regarding packet delivery and network performance. In contrast, the proactive approach incurs higher overhead in managing policy changes. The remaining sections of the paper are organized as follows. "Related work" provides an overview of related work and its limitations. "Problem formulation and proposed solution" explains the problem formulation and introduces the proposed solution to address the challenges. “Performance evaluation” includes a comprehensive performance evaluation, utilizing simulation results to assess the effectiveness of the proposed solution. Finally, “Conclusion and future work” concludes the paper by discussing the contributions and future research directions are provided to carry forward this research.

## Related work

This section describes studies related to flow rule installation mechanisms and discussion on ACL policy implementation in SDN environments. In^21^, an Efficient Policy Enforcement (EPE) scheme is presented which states that change in ACL policies can result in packet violations associated with flows that already have flow rules installed at the data plane. The proposed approach aims to identify the controller's policy changes and remove any conflicting flow rules previously installed at the SDN switches. However, it is noted that this approach exhibits a longer detection time for policy change, leading to an increased number of packets that violate the ACL policy. In^22^, Graph-Based Policy Enforcement (GPE) is proposed for reactive flow rules installation. It can detect changes in ACL policy by utilizing graph matching with less time to avoid packet violations and network inefficiencies. OpenFlow Rules Placement Problem (ORPP)^23,24^ proposes two proposals for online and offline flow rule placement in SDN. OFFICER is the first proposal that provides optimization techniques to install flow rules as per ACL policy requirements which are known and stable over a period. The other one is aOFFICER, which is based on adaptive control to install flow rules, for example, load balancing, where flow rules are unknown and unstable. In^25^, the authors propose a proactive approach for installing flow rules in the context of efficient communication within the Internet of Things (IoT). This approach addresses two main challenges: the delay in flow rule installation and congestion resulting from packet-in messages. By adopting this proactive strategy, the proposed approach mitigates these challenges effectively. It not only reduces the installation delay but also helps alleviate congestion issues. Additionally, this proactive approach contributes to energy savings and optimizes the utilization of network node resources in IoT environments.

Flow rules in SDN can be implemented in switches through two methods, i.e., wildcard matching and exact matching. Wildcard matching increases the flow rules’ reusability and decreases the occurrence of packet-in messages. Conversely, with exact matching, a packet-in message is generated for almost every flow passing through the switch, consuming valuable resources. To tackle this challenge, researchers propose employing a load balancing mechanism through the proactive flow rules installation on several switches or reactive installation of flow rules within every switch. In^26^, the proposed technique installs flow rules in SDN switches prior to packet forwarding. In certain scenarios within SDN, the succeeding packets arriving at switches without matching flow rules may result in packet discards. The proposed technique computes the packet's arrival delay and the flow rule installation time to address this issue. Suppose a delay exists between flow rule installation and packet arrival. In that case, the technique introduces an appropriate delay to the packet at the predecessor switch to ensure synchronized availability of flow rules. Utilizing the Pyretic programming language, PyResonance^27^ creates state-based ACL policies. Pyretic's composition operators can create complicated network control programs by combining its modular control programs. Additionally, SDN utilizes composition operators to define state-based policies, enabling the combination of diverse tasks through a finite state machine and accompanying network application. By composing multiple tasks, each including a finite state machine and a matching network program that dictates forwarding behavior for that state, these composition operators enable network operators to design state-based network policies. A reverse update approach for SDN is proposed in^28^, which ensures the preservation of flow attributes during the transition period. It suggests a formal model based on the idea that in-transit packets are always handled by the same or a more recent policy in the subsequent forwarding hops to achieve consistent policy updates. Additionally, it offers a consistent and effective policy update technique and a relaxation notion for per-packet consistency in the data plane. In communication, networks often have link failures, which influence network performance.

In^29^, a Reliability Aware Multiple Path Flow Rule (RAF) solution is proposed, which evaluates link reliability depending on the reliability value of the principal path and installs minimum flow rules following ACL regulations and network topology for multiple paths. In^30^, the authors provided a comprehensive overview of the implementation of ACL policies in IoE environments. In SDN, the ACL policies are declared at the controller that computes the path and flow rules between source and destination regardless of whether the hosts in an ACL policy are active. In this way, the controller experiences a longer processing delay, increasing the data packets' end-to-end latency. The research work in^31^ resolves the problem of matching the controller's packet with active ACL policies only, resulting in reduced data packet travel time from the source to the destination and processing time at the controller. Additionally, it increases the scalability of the controller by reducing the additional load of inactive hosts, thereby improving the allocation of the controller's precious resources for other important tasks. In^32^ a systematic design strategy is proposed to apply ACL policies optimally utilizing decision trees and K-partite graphs to overcome these difficulties. The best location to apply the ACL policies according to the specified criteria is then found by traversing the K-partite graph. The simulation findings suggest that the proposed method performs better, which increases network efficiency.

The authors in^33^ examine SDN administration and control actions regarding to WSNs. They explored how to improve the flexibility, manageability, and overall performance of WSNs by utilizing SDN. The authors emphasized implementing ACL policies in WSN by utilizing the SDN. SDN offers a more practical network paradigm to implement ACL policies as compared to traditional networks. Most ACL policy implementation studies use a reactive manner, showing that the latest policies cannot take effect immediately, resulting in network inefficiencies. The research in^34^ introduces an SDN-based MLS-Enforcer controller program, which offers secure network node relabeling in response to dynamic topology conditions and network traffic requirements. Additionally, it efficiently deploys network-level Multilevel Security (MLS) regulations to ensure effective security measures in the network environment.

Research work in^35^ focuses on detecting anomalies in network policies before deployment, ensuring verification before installing flow rules in the data plane. The approach involves formally representing forwarding policies and detecting anomalies against the corresponding set of flow rules. Additionally, network administrators have the flexibility to define their own anomalies. In^36^, a novel endogenous network access control mechanism is presented, which utilizes a reconfigurable decision tree model. The proposed mechanism addresses the limitations of static and repetitive traditional access control systems. Initially, it examines the optimal deployment of access control policies by selecting a decision tree model that aligns with the network architecture. The authorization decision process, traditionally executed on the control plane, is then transferred to the forwarding device in the data plane to minimize processing delay. The results demonstrate that the proposed approach achieves access control with lower memory usage, increased accuracy, and less impact on forwarding devices. The research work in^37^ presents an SDN-based IoT system against various cyber threats. The proposed solution consists of a Policy-Enforcement Module (PEM) that mitigates attacks and Binary/Multi-class Classification Modules that identify potential threats in the IoT environment. It utilizes different machine learning techniques to solve the classification problems and compare their effectiveness. In this paper, the existing studies are analyzed and discussed related to the research problem of efficiently handling ACL policy change in SDN.

The research work presented in^38^ introduces a robust security policy enforcement mechanism that offers a standardized set of intent rules, effectively shielding the network administrator from the intricacies of the SDN controller's format. Furthermore, this mechanism significantly alleviates the complexities associated with checking flow rules conflicts at the data plane. By leveraging network service function chaining (SFC) for inter-domain policy administration, the proposed mechanism demonstrates its deployability across multiple SDN domains and hybrid networks, extending its reach beyond a single SDN domain. In^39^, the research presents an innovative approach to resolve the challenge of optimizing the placement of ACL rules within the TCAMs of switches. The proposed approach specifically targets the achievement of appropriate ACL match-action and management, effectively eliminating the trade-off between scalability and rule management latency while enhancing TCAM efficiency. By utilizing this approach, network administrators can balance the number of ACL rules accommodated within the TCAMs and the responsiveness of rule management operations. Consequently, the overall efficiency of the TCAMs is significantly enhanced, leading to improved network performance and effective utilization of network resources.

The research work described in^40^ presents a system model of security policy (SPM) for SDN networks, offering an automated approach to transform security policies into corresponding flow entries. This model incorporates multiple modules such as security policy, topology discovery, runtime monitoring, path generation, and flow entry generation. By utilizing these modules in a coordinated manner, the proposed approach computes the necessary flow entries. It installs them on the appropriate paths within the data plane, aligning with the defined security policy. This automation eliminates manual configuration and reduces the likelihood of errors, ensuring consistent and efficient enforcement of security policies throughout the SDN network. In^41^, the PrePass-Flow system introduces an innovative approach to enhance network reliability by incorporating a predictive technique to anticipate and prevent connection failures. The system effectively mitigates potential link losses by recalculating the locations of ACL policies and proactively applying flow rules based on the updated locations. This method significantly reduces packet violations and network reachability problems that may arise due to link failures. Additionally, this approach ensures that ACL policies are dynamically updated to maintain optimal efficiency and dependability, improving overall network performance and minimizing disruptions caused by link failures. The implementation of PrePass-Flow brings enhanced stability and operational continuity to network environments.

The existing studies are based on different problems, like violations and incorrect implementation of ACL policies, forwarding loops, black holes, flow table consistencies, network topology change, etc. in various kinds of implementations in SDN. The violation and incorrect implementation of ACL policies are high-priority invariants due to misconfigurations, conflicts, overlapping, and mistranslations that may result in packet violations, network attacks, and reduced network efficiency. ACL policies are widely used in SDN for regulating access to network resources. The correct implementation of ACL policies increases network security by preventing unauthorized access, simplifying network management, and efficient resource utilization by allowing only necessary traffic. However, configuring and maintaining ACL policies can be time-consuming and prone to errors. The existing studies discussed mechanisms that are helpful to implement ACL policies in SDN. Despite the advancements in various mechanisms discussed, they often fail to detect ACL policy changes and manage conflicting flow rules effectively. Consequently, packet violations and network inefficiencies can arise. Furthermore, there is a lack of comprehensive analysis on the proactive and reactive flow rule installation approaches specifically in relation to policy changes. The evaluation of packet violations and network inefficiencies in such scenarios has not been extensively explored before. These gaps highlight the need for further research and development to address these challenges and enhance network systems' overall performance and adaptability.

## Problem formulation and proposed solution

ACL policies change over time in communication networks due to application/user requirements or network topology modifications. This change in ACL policies may result in overlapping or conflicting access to network resources because the network applications need to access data from diverse application platforms, servers, clouds, etc. To solve these problems, composing and formalising the ACL policies is necessary to prevent conflicts or overlaps in accessing network resources. The ACL policies are represented using a 5-tuple < Source, Destination, Protocol, Port, Action > . Here, Source and Destination consist of multiple subsets of IP Addresses. The Protocol includes Transmission Control Protocol (TCP) or User Datagram Protocol (UDP). The ports include destination port numbers for the applications and the Action field contains options for “Permit” or “Deny”. These policies are then transformed into a policy implementation file and transmitted to the controller for further processing. The controller plays a crucial role in computing flow rules based on the network topology and policies when a flow is established. These flow rules are stored in a hash table data structure and installed at the switches along the shortest path determined by the controller. A consistent hash function ensures the same output for a given input during hash function execution, thereby enabling accurate tracking of specific flow rules associated with defined ACL policies. If a policy change occurs, the controller employs the proposed techniques based on matrices^21^ and graph matching^22^ to detect the change in ACL policies. These approaches translate policies and their components in vector and graph form respectively. This translation helps to understand the reachability model expressed by the ACL policies and makes it easy to compare with similar translation of new, updated, or refactored policies. If no policy change is detected using the mentioned approaches, no action is taken, as the network is already operating according to the most recent policies.

**Figure 3:**
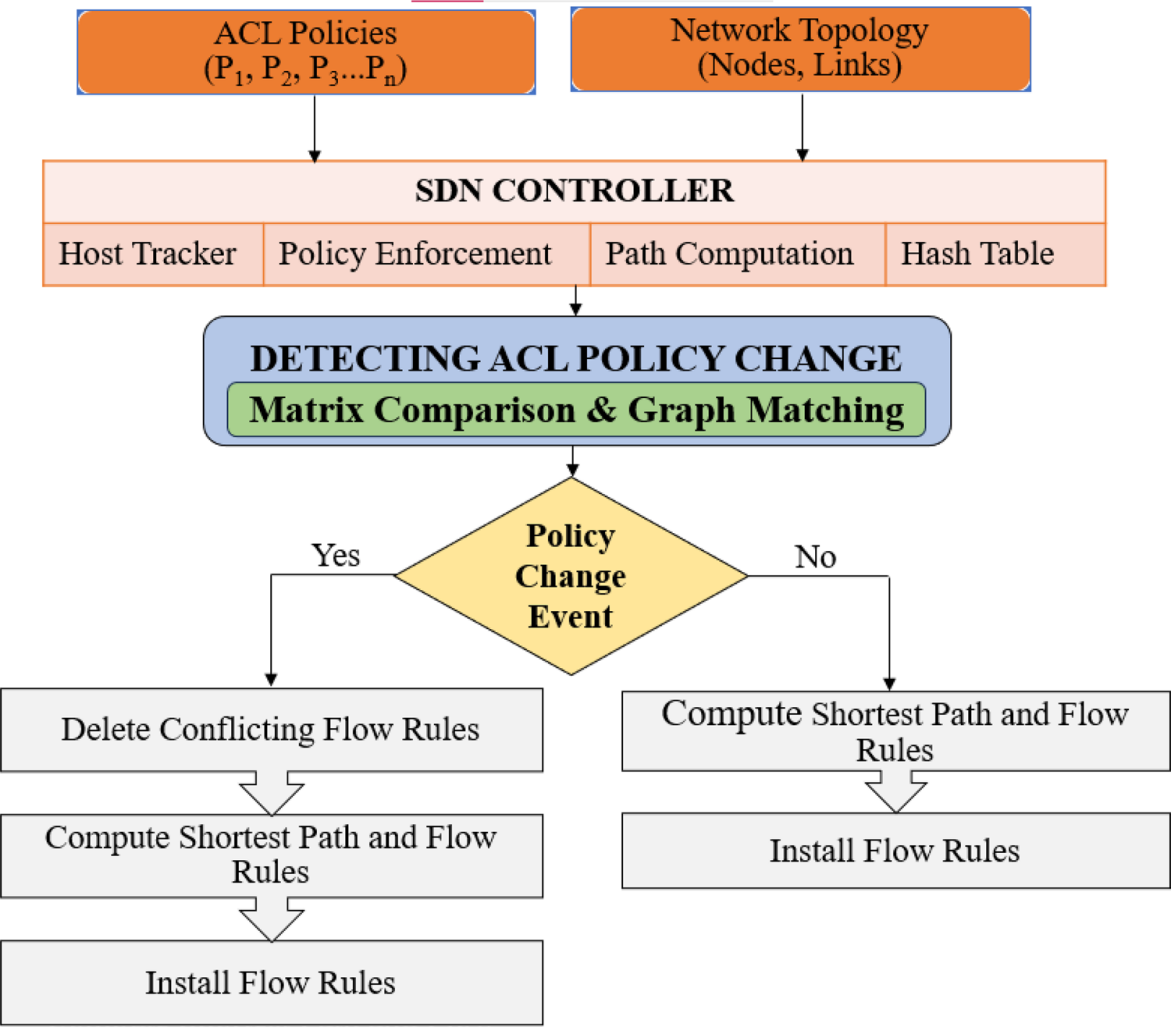
ACL policy change implementation proposed solution.

The controller employs two methods for the flow rules installation. In case of the proactive flow rule installation, it starts the policy change localization procedures for each new incoming ACL policy. If it is found that the new ACL policy is conflicting and can incur misconfiguration, the controller first deletes the conflicting rules installed in switches. In the reactive policy installation, the controller detects the policy change after its deployment. This procedure is more complex than the proactive approach because the policy change identification starts when multiple flow rules are available in switches to treat the single packet^42^. Conflicting flow rules are identified if a match is found, while non-conflicting flow rules indicate the addition of new policies in both proactive and reactive cases. In the absence of conflicts, new policies are added seamlessly. On the other hand, if conflicts arise, the proposed method based on the graph and matrix matching approaches decide to remove all flow rules that do not align with the modified ACL policies from both the controller's cache and the flow tables of the switches. The shortest path and new flow rules are then computed based on the updated ACL policies, installed at the switches, and cached in the controller. The overall proposed solution is depicted in Fig. 3.

This paper formulated a state-of-the-art model for the ACL policy change implementation mechanism. This is the ultimate in finding the optimal performance of the pure SDN integration in the emerging architecture of the network services. More concretely, the demand for end-user devices is in flux and more stringent with time. This makes the network operators consider the multiple dynamic states of the network traffic flow. The SDN as a promising solution needs to evaluate a couple of aspects in its proactive and reactive approaches of resources, services, and control applications (i.e., protocols). For this reason, the policy change abstraction of SDN is modeled in proactive and reactive installation. This approach has the potential to contribute to policy change management in 5G and beyond SDN-managed domains. , It is also beneficial for the SDN Integrated vertical use cases like Internet of Medical Things and Massive IoTs^43^ where the overhead of network reachability change management should be minimal. Network reachability and dynamic change management are, safe to say, a complex problem that needs to be analyzed first for the impact of the policy in the network. For example, conflicting policies installed at various time instances can initiate quality-of-service concerns among the multiple tenants connected via SDN software control functions. This impact can be observed for a single production packet in the network with suspicious status by policy conflicts in SDN. The proposed solution is formulated considering the performance parameters, specifically the overhead of ACL policy changes. The working of the proposed solution is presented in Algorithm 1 and the details of functions and modules are explained in Table 1 for better understanding.

**Algorithm 1:**
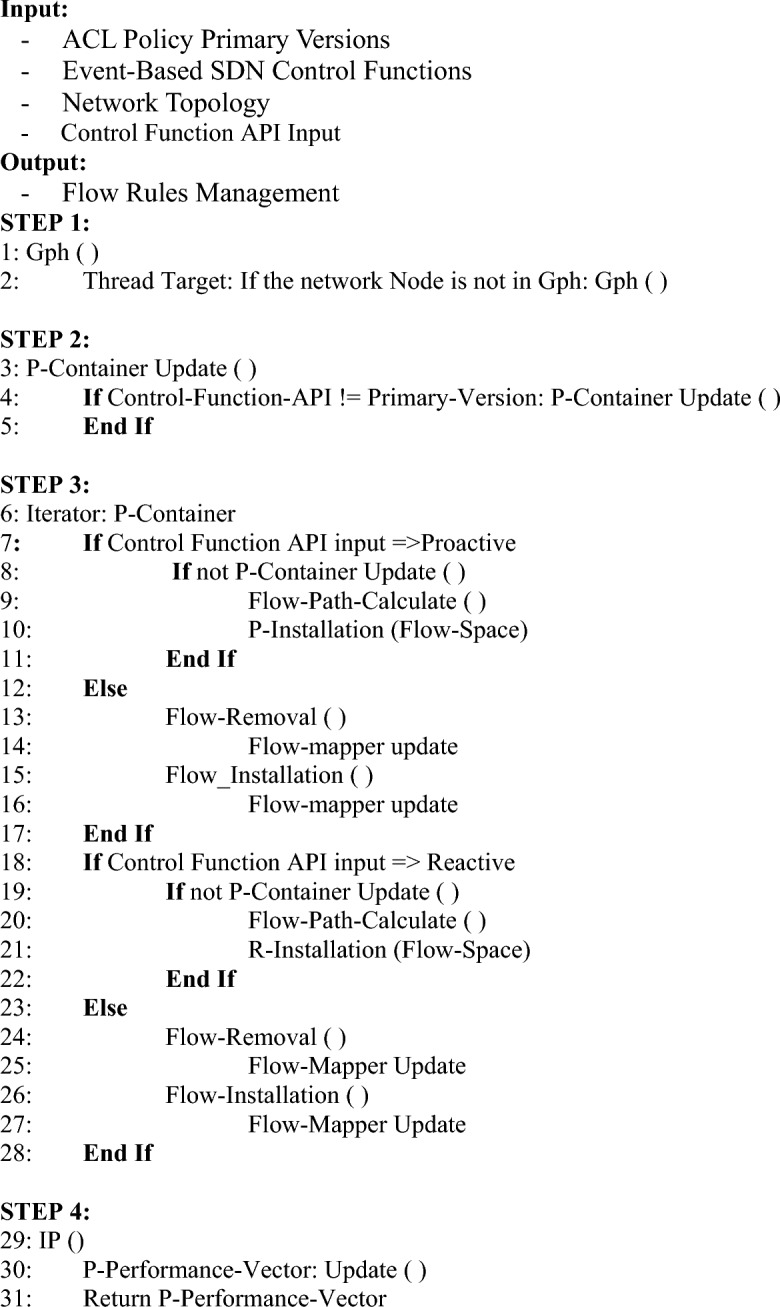
ACL policy change implementation.

**Table 1:** Problem formulation and proposed solution.

Symbols	Explanation
P-container	ACL policy is stored in a multidimensional hash table data structure. We can say that the components of the policy are close to a URLLC integrated network policy. For example, this P-container can have various party identifications, such as organizational tenants, edge network subnets or symmetry of SDN controller identifications, or even Roadside Units (RSU) identification in case the policy change environment is the Internet of Vehicles. Similarly, the ports of the protocol and applications facilitate bidirectional communication among different network objects
Gph	Gph is the network topology virtual graph that dictates all the network linkage properties by subscribing to the SDN programmable functions. In case the network topology changes, the function waits to update the network graph. Additionally, the edges in Gph hold properties of a network link, such as link capacity and historical statistics in the form of nested vectors. These properties help the SDN control functions to make decisions about the path
Flow-path-calculate	This function is employed for calculating the flow path calculation from the source subnet to the destination subnet using the Dijkstra algorithm for this purpose. The Algorithm is presented in Algorithm 2
Path-space	Path-space is the set of all communication paths among the communication parties
ith-path	It is a specific path to test for a corresponding policy change proactively or reactively
P-installation	This function performs the proactive flow rule installation. It installs flow rules proactively in advance before communication as per the ACL policies and network topology between source and destination on the shortest path. The algorithm for R-Installation is presented in Algorithm 3
R-installation	The function performs the reactive flow rule installation. It installs flow rules reactively in response to the Packet-In message at the controller. The algorithm for R-installation is presented in Algorithm 4
P-change	It denotes the event of ACL policy change where the control function API triggers an event to alter the traffic behavior
P-version	This denotes the version of a policy. The default version of a policy is the primary policy in our simulations. It also denotes the early intents of a network administrator regarding service reachability configurations
P-version-map	This represents each policy version (P-version) in a multidimensional P-container. The multidimensionality helps to accommodate the components of a P-version for instance, port or protocol
P-Performance-Vector	The corresponding performance vector (i.e., impact on latency while changing several policies)
Flow-mapper	Flow-mapper keeps records of the flow installed with timestamps, the number of flow rule entries on the switch, control function execution time, pair of IP addresses, and flow residing time in the intermediate communication OpenFlow-enabled switches
Flow-rule-installation	This function triggers the events of control network traffic over TCP or UDP for the installation of flow rules that correspond to a certain policy. It incurs control traffic between the controller and OpenFlow-enabled forwarding devices
Flow-rule-removal	This function employs the OpenFlow API (specification 1.3) command to remove the installed flow entries from the switches. This flow removal is after the computation and calibrates the impact on performance parameters of new specific policy versions, which are introduced or exposed during the recursive iteration of the P-container
Control-function -API	Control function API in this model is used to orchestrate the above-specified functions. This API works with the POX event handling approach and python supported multithreading, and semaphores to keep the cohesion and balance the coupling of network software functions. The Control_Function_API_Input comes with the flexibility of either a proactive or reactive flow rule installation, specific to the interest of a network administrator in a domain or segment of the network
IP ()	Its function is to generate IP traffic among the tenants of the target policy communication. This function is composed of further methods like socket programming (i.e., client/server-side script utilizing the TCP, UDP protocol to generate the traffic between communicating IPs)

The proposed approach works for the abstraction of Proof of Work. There are two modes of flow rule installation which are governed by the control API. In the case of the proactive installation, all the flow rules in the Path-Space are iterated and installed. The proactive flow rules installation is performed by using Algorithm 3. While in the case of the reactive, the controller computes only the ith path (an alternative path to disrupted ones) among the communication parties. Suppose a new version of the reachability map or a single policy is received. In that case, the controller may have the option to compute multiple paths at once (proactive) or reactively install a single flow path. Each new version of the policy is desired to install with the least latency regardless of the mode of the controller installation. The algorithm updates the performance vector iteratively. It can be safely claimed that the continuity of vector updates is a solution to gauge the performance of SDN use cases, as the innovations and communication model ingredients continue to update. The reactive flow rules installation mechanism is performed by using Algorithm 4

**Algorithm 2:**
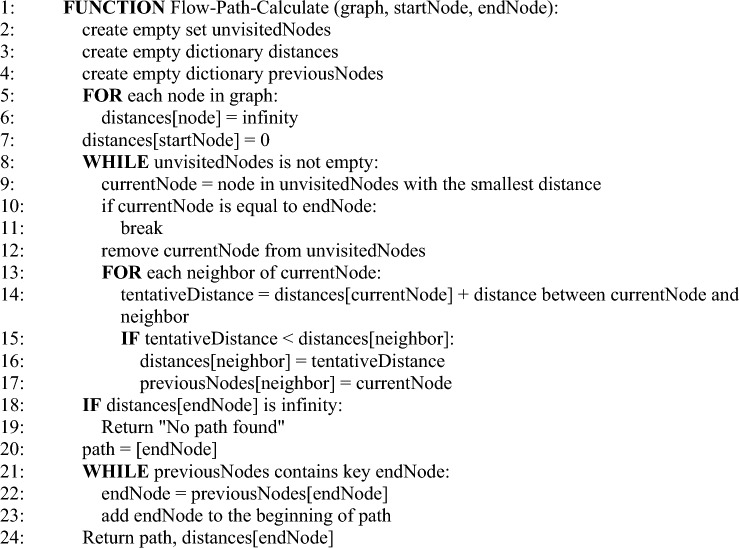
Flow path calculation.

**Algorithm 3:**
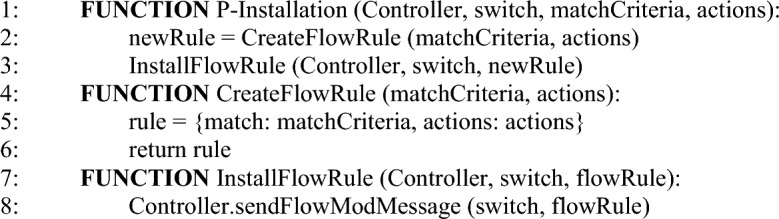
Proactive flow rule installation.

**Algorithm 4:**
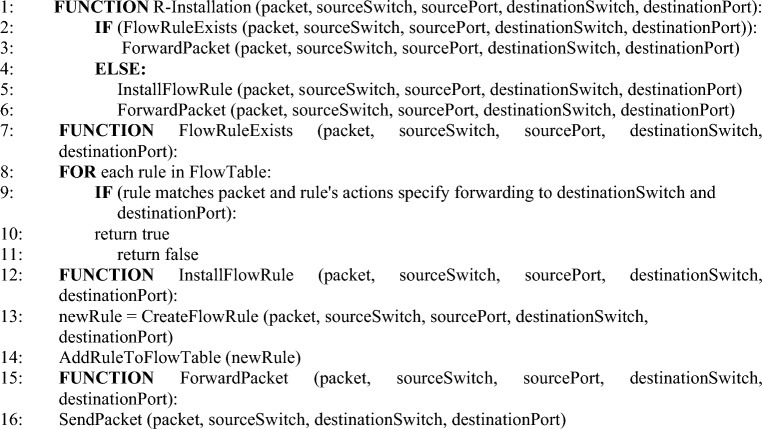
Reactive flow rule installation.

.

## Performance evaluation

In this research work, Mininet SDN Network Emulator and POX SDN controller are chosen to analyze the performance of our proposed solution. This is a widely used simulator in the research community due to its various advantages, for instance, it is an easy interface to the remote SDN controller on top of TCP or UDP protocols. This research utilizes an HP Probook 450 G5 laptop equipped with an Intel Core i5-8250U CPU clocked at 1.60 GHz (8 CPUs), 16 gigabytes of RAM, and 500 gigabytes of solid-state drive. The laptop runs the Linux operating system, specifically Ubuntu 16.04. The proposed application is written in Python to be executed on the POX controller to analyze the proposed solution. The network topology is created using the Mininet 2.2 EEL simulator to analyze this research. The topology includes the configuration of hosts, links, open-flow soft switches (specifically OVS 2.5.2), which support OpenFlow versions 1.3 and higher. The POX SDN controller is used to control and manage the network. In this paper, the real time network topology and ACL policies of an educational institute is utilized for fair analysis. The network topology comprises 9 OpenFlow soft switches and 30 hosts that can communicate using a central SDN POX controller based on TCP or UDP protocols. Additionally, a range of 1,000 to 80,000 packets are randomly transmitted from diverse sources to multiple destinations. The ACL policies are replica of a campus network and are functional for communicating different zones and sectors on the campus. The total simulation time of experimental evaluation in a single epoch is set to 100 s while an interval or time instance to repeat recursion before the end of the simulation is selected randomly for the fair analysis of results. Finally, the comparison of proactive and reactive flow rules installation is presented by utilizing our EPE^5^ approach and GPE^6^ approach by using the below performance metrics, by varying frequency of policy change, inter-packet delay, and flow timeout value.

- (a) Packet violation percentage
  Packet violation percentage (PVP) is determined by calculating the ratio of the total number of packet violations to the total number of transmitted packets. It measures the percentage of packets that do not adhere to the defined policy.
  $$\text{PVP}=[ \frac{\text{Total number of packet violations}}{\text{Total number of transmitted packets}}] *100$$
- Successful packet delivery
  Successful packet delivery (SPD) is determined by calculating the ratio of the total number of successfully delivered packets as per policy to the total number of transmitted packets. This metric measures the percentage of packets that reach their intended destinations in accordance with the specified policy.
  $$\text{SPD}= [\frac{\text{Total number of successfully received packets}}{\text{Total number of transmitted packets}}] *100$$
- Normalized overhead
  The normalized overhead (NOH) is computed by dividing the total number of transmitted packets by the total number successfully received packets as per policy. This metric helps evaluate the traffic overhead by considering the number of packets transmitted relative to the number of packets successfully delivered according to the policy.
  $$\text{NOH}=[ \frac{\text{Total number of transmitted packets}}{\text{Total number of successfully received packets}}] *100$$
- Network throughput
  Network throughput (NTP) is measured as the rate at which packets are successfully delivered to the destination nodes in accordance with the policy. It represents the number of packets per second that are effectively transmitted and received within the network, reflecting the network's overall capacity to handle and deliver packets in a timely manner.
  $$\text{NTP}= \frac{\text{Total number of successfully received packets}*8*\text{packet size}}{\text{Time Duration in number of seconds of simulation}}$$
- Average end-to-end delay
  Average end-to-end delay (AED) is the time duration required for a packet to travel from the source host to the destination host. We calculate the average end-to-end delay for all successfully received packets according to the ACL policy.
- Policy change detection time
  Policy change detection time (PCDT) is the duration of time that it takes the controller to identify changes in ACL policies, delete conflicting flow rules, and install new flow rules at the data plane.

### Simulation results by varying frequency of ACL policy change

**Figure 4:**
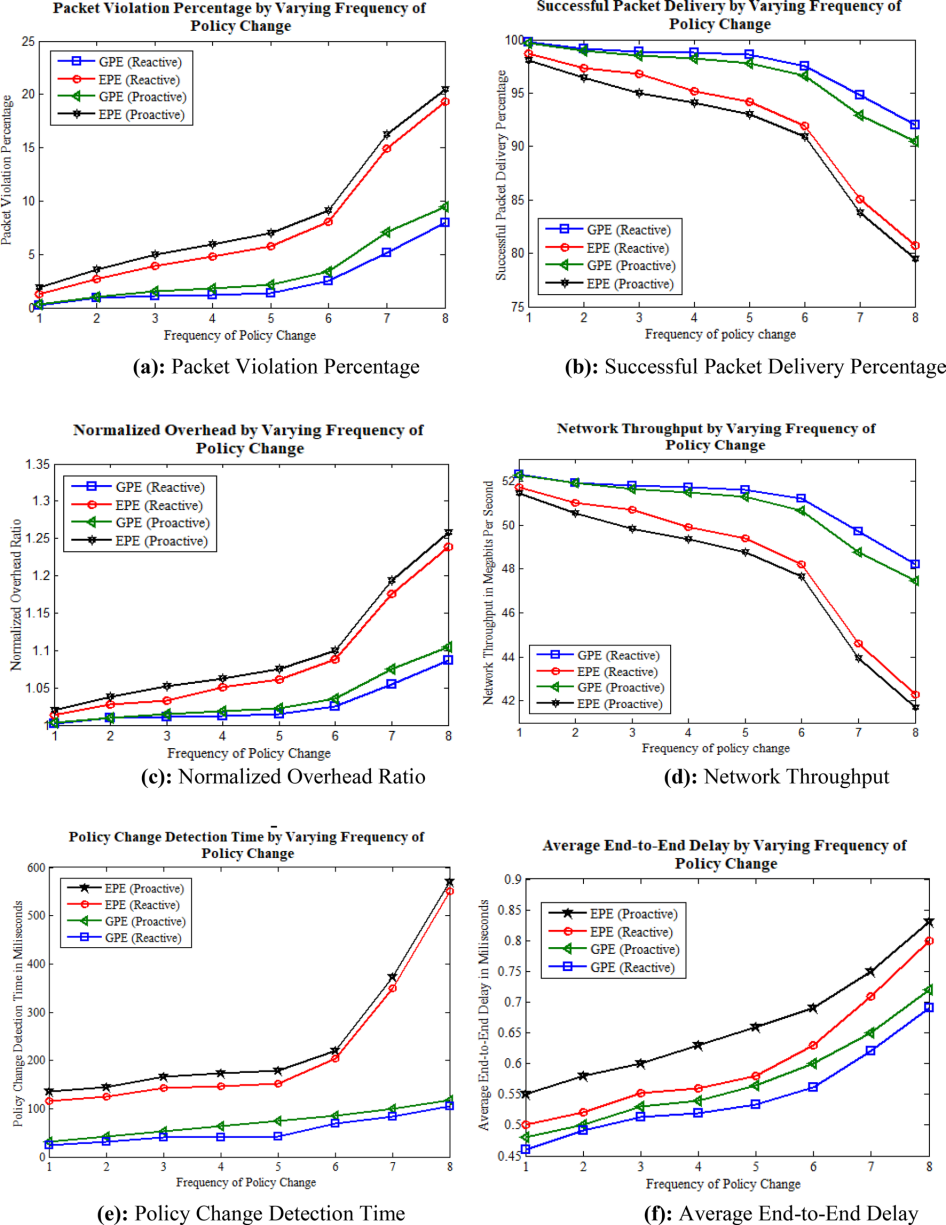
Simulation results by varying ACL policy at different time instances. **(a)** Packet violation percentage, **(b)** successful packet delivery percentage, **(c)** normalized overhead ratio, **(d)** network throughput, **(e)** policy change detection time, **(f)** average end-to-end delay.

The simulation results presented in this study are derived by utilizing static and dynamic parameters. The static parameters encompass the default flow timeout value and the inter-packet delay of 0.6 ms (ms). On the other hand, the dynamic parameter considered is the frequency of policy change, specifically selected as 1, 2, 3, 4, 5, 6, 7, and 8 policies, which are randomly altered during the simulation. The simulation was executed by employing these specified parameters, and the obtained outcomes are depicted in Fig. 4.

The results show significant packet violations in the proactive approach as compared to the reactive approach as illustrated in Fig. 4a in both GPE and EPE based ACL policy implementation mechanisms. This is because GPE demonstrates superior computational efficiency, leveraging optimized graph traversal for the rapid identification of modifications in ACL policies. In large and complex environments, GPE becomes more efficient compared to EPE, which may incur higher computational costs and longer detection times due to its inherent structural limitations. In a proactive approach, the change in ACL policies significantly impacts the forwarding devices, which require increased control transmission tasks such as installing, deleting, or modifying existing flow rules. Specifically, placing nth order flow rules involve computing and deploying flow rule entries for all source and destination pairs of a particular ACL policy. It impose substantial resource requirements on those switches which utilize proactive flow rule installation mechanisms. As a result, it leads to higher resource utilization and potentially impact the overall performance. The proactive approach may also require suspending network-provided services at discrete-time intervals to handle the exponential influx of ACL policies. However, the reactive approach typically involves fewer computations in the event of an ACL policy change, as it needs to handle a smaller number of flow rules at the switches. In some cases, it may only be necessary to append new flow rules on-demand, which reduces the computational overhead compared to the proactive approach. Similarly, both successful packet delivery and network throughput increases with the less frequency of ACL policy change. This impact can be seen relatively greater in proactive approach due to the more computations as compared to the reactive appraoch as shown in Fig. 4b–d.

The normalized overhead also increases in the case of proactive flow rule installation as compared to reactive approach, as shown in Fig. 4c. This is because the proactive approach requires the computation, deletion, and installation of additional flow rules in advance, even if they are not utilized for communication. This extra overhead becomes particularly significant when many flow rules are involved. The results state that the ACL policy change detection time in GPE approach remains relatively stable and efficient, however, it fluctuates at higher side in case of EPE approach as shown in Fig. 4e. In addition, in proactive approaches where rules are pre-installed, the detection process involves identifying conflicts between existing and new flow rules upon policy change event. As the frequency of policy changes escalates, proactive approaches experience increased computational overhead and, subsequently longer detection times due to increased rule evaluations and replacements. Finally, the average end-to-end delay is less in the case of reactive approaches than the proactive as shown in Fig. 4f. In conclusion, when assessing the frequency of policy change as a dynamic parameter, the technical aspects governing policy change detection time in reactive and proactive approaches significantly diverge based on their core operational mechanisms, the optimization methodologies employed, and the computational demands entailed in continuous monitoring and rule adaptation. The distinctiveness between these approaches lies in their response mechanisms to policy alterations, where reactive systems rely on immediate detection upon change occurrence, potentially showing stability until overload especially in case of graph based approaches. However, in proactive systems, with pre-installed rules, face challenges in swiftly identifying conflicts between existing and new flow rules, leading to increased computational overhead and potentially prolonged detection times, particularly with escalated policy change frequencies.

### Simulation results by varying inter-packet delay

**Figure 5:**
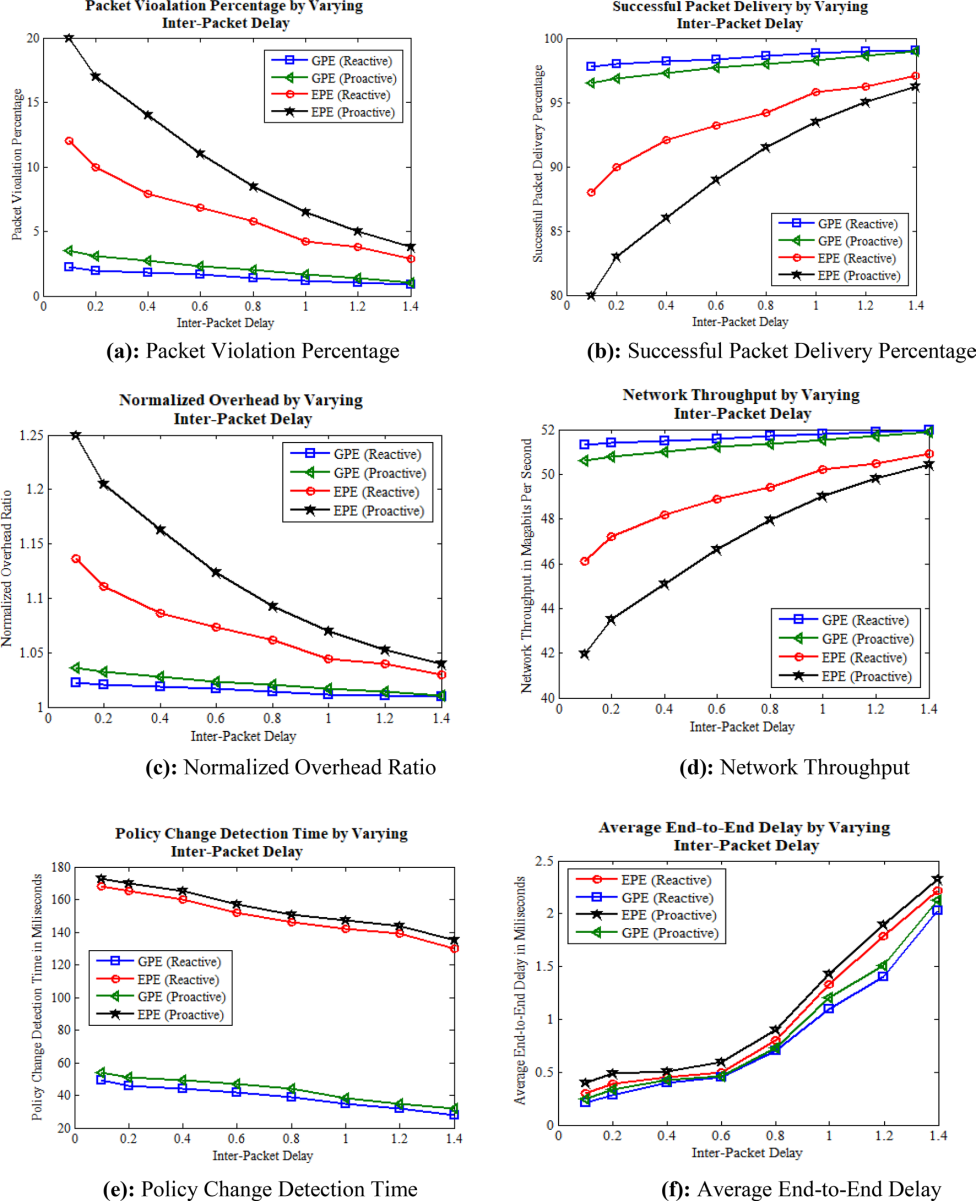
Simulation results by varying inter packet delay. **(a)** Packet violation percentage, **(b)** successful packet delivery percentage, **(c)** normalized overhead ratio, **(d)** network throughput, **(e)** policy change detection time, **(f)** average end-to-end delay.

To examine the impact of the inter-packet delay, the static parameters in the simulation include a fixed frequency of policy changes set to 5, along with default flow rule timeout values. The dynamic parameter chosen is the inter-packet delay, which is set to values of 0.1, 0.2, 0.3, 0.4, 0.5, 0.6, 0.7, and 0.8 ms. Inter-packet delay refers to the time interval between the transmissions of consecutive packets in a network communication scenario. This parameter signifies that our application generates a random number of packets per second with a specified delay in milliseconds. If the delay is lower, more packets per second are transmitted; conversely, an increase in delay results in a reduction in the number of transmitted packets generated by the application. By implementing these parameters, the simulation was conducted, and the corresponding results are presented in Fig. 5.

Figure 5 depicts simulation results obtained by varying the inter-packet delay. The results reveal that increasing the inter-packet delay reduces packet violations due to the decreased number of packets transmitted per unit time between the source and destination. This effect is particularly noticeable in the case of reactive flow rule installation compared to proactive flow rule installation, as illustrated in Fig. 5a. This reduction in packet violations stem from the fact that the reactive approach efficiently deletes existing conflicting flow rules as compared to the proactive approach. This trend is consistent in GPE-based implementation, where the percentage of packet violations is significantly lower than in EPE-based implementation. This difference is attributed to the GPE approach's ability to promptly detect ACL policy changes, leading to the earlier deletion of outdated flow rules and mitigating packet violations. Additionally, successful packet delivery and network throughput increases with the increase in inter-packet delay in both reactive and proactive scenarios. Notably, Fig. 5b–d demonstrate that reactive flow rule installation outperforms proactive flow rule installation. The normalized overhead ratio also favors the reactive approach due to the reduced number of flow rule installations, deletions, and modifications compared to proactive flow rule installation, as depicted in Fig. 5c. This outcome emphasizes the operational efficiency of the reactive approach in managing the overhead associated with flow rule operations.

The ACL policy change detection time is influenced by inter-packet delay in both reactive and proactive approaches. As illustrated in Fig. 5e, higher inter-packet delays facilitate faster policy change detection, allowing for more prompt identification of changes, and vice versa. These findings suggest that GPE outperforms EPE in detecting ACL policy changes, primarily because of GPE's computational efficiency, optimized graph traversal, and swift identification of modifications. Furthermore, changes in inter-packet delay also affect end-to-end delay, as depicted in Fig. 5f. A higher inter-packet delay exacerbates end-to-end delay by prolonging latency, queueing delay, and idle time, while a lower delay mitigates these factors, thereby reducing overall end-to-end delays. Notably, GPE demonstrates superior efficiency in detecting ACL policy changes, resulting in fewer delays compared to EPE, which requires more time for computation and change detection, especially in dynamic environments. Additionally, within an SDN environment, the impact of inter-packet delay on end-to-end delay varies depending on the flow rule installation mechanisms. Specifically, while both reactive and proactive mechanisms are utilized, the reactive approach often outperforms proactive installation due to its ability to dynamically respond to network events, thus optimizing packet forwarding and minimizing delay effects.

### Simulation results by varying timeout value of flow rules

**Figure 6:**
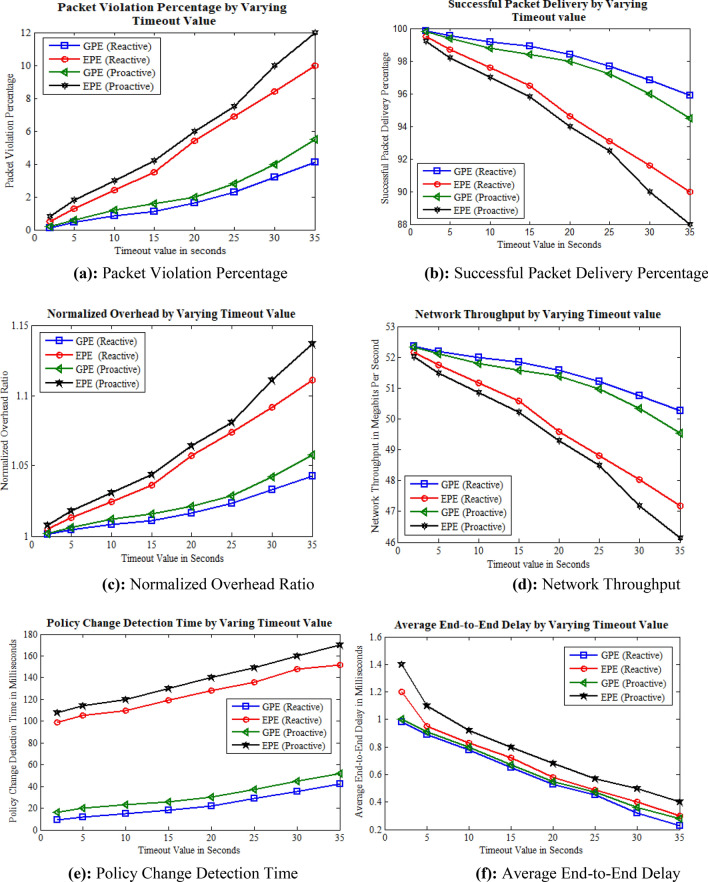
Simulation results by varying timeout value. **(a)** Packet violation percentage, **(b)** successful packet delivery percentage, **(c)** normalized overhead ratio, **(d)** network throughput, **(e)** policy change detection time, **(f)** average end-to-end delay.

The simulation was conducted to analyze the effects of varying the timeout value of flow rules with the following static parameters: a frequency of policy change set to 5, a inter-packet delay of 0.6 ms, and a dynamic parameter of the flow rule timeout value. The flow rule timeout value was randomly assigned values of 2, 5, 10, 15, 20, 25, 30, and 35 s. The simulation was executed based on these parameters, and the corresponding results are presented in Fig. 6.

The study explored the impact of varied timeout values of flow rules on packet violations, focusing on proactive and reactive flow rule installation mechanisms while utilizing GPE and EPE for ACL policy change detection. As shown in Fig. 6, the results reveal a direct correlation between incremental timeout values and an increase in packet violations. In the proactive approach, this correlation arises from the prolonged presence of flow rules within switch flow tables, leading to advanced flow rule installation. Consequently, there is a higher occurrence of packet violations compared to the reactive mechanism, where flow rules are pre-installed on the data plane. Furthermore, the proactive approach leads to an extended lookup time for flow rules during policy alterations, primarily attributed to the increased deletion of old rules and the installation of new flow rules. These factors cumulatively contribute to a higher incidence of packet violations in the proactive approach compared to the reactive approach, as illustrated in Fig. 6a. However, it is notable that in the case of GPE, packet violations are reduced due to its efficient ACL policy change detection, as opposed to EPE where the policy change detection time is prolonged. This extended detection time in EPE results in the deletion of old flow rules and the installation of new flow rules taking more time, contributing to a higher frequency of packet violations. Figure 6b–d present the simulation results pertaining to the successful packet delivery percentage and network throughput. Notably, the GPE approach with reactive flow rule installation demonstrates the highest network throughput and successful packet delivery. This superiority can be attributed to GPE's efficient ACL policy change detection, coupled with the reactive mechanism that installs only the necessary flow rules between the source and destination. In contrast, both the proactive approach and the EPE mechanism exhibit comparatively lower network throughput. The proactive nature of flow rule installation, characterized by the pre-emptive addition and deletion of numerous flow rules, contributes to an increased normalized overhead. This, in turn, leads to decreased network throughput as observed in the simulation results. Additionally, an increase in the flow timeout value, causing flow rules to persist in the flow tables of switches for longer durations, has a more pronounced impact on both network throughput and successful packet delivery during ACL policy changes.

The findings reveal that the proactive approach exhibits a significantly higher normalized overhead ratio compared to the reactive approach in both GPE and EPE strategies, as depicted in Fig. 6c. However, with GPE, the normalized overhead remains lower due to its efficient policy change detection nature. This disparity stems from the proactive nature of adding all flow rules in advance. Within the proactive method, situations may arise where flow rules are installed but remain unused during communication, potentially leading to a higher normalized overhead. The ACL policy change detection is influenced by timeout values, as demonstrated in Fig. 6e. With proactive mechanism, the detection time significantly increases due to the computational demands associated with pre-installing flow rules, which includes the identification of conflicting flow rules and the installation of new ones to align with modified ACL policies. Notably, with GPE, the change detection time consistently remains less, yielding superior results. However, matrix-based approaches, such as EPE, may necessitate more effort to adapt to changes in ACL policies might be more time-consuming and error-prone. Figure 6f illustrates that varying timeout values impact end-to-end delay in both reactive and proactive flow rule installations. Small timeout values lead to quicker flow rule changes, resulting in more end-to-end delays due to installing more flow rules. In addition, the higher timeout values prolong the flow rules installation within switches' flow tables, intensifying computations required for ACL policy implementation, conflict detection among flow rules, and generating new rules. This computational load is notably amplified in the proactive approach compared to the reactive approach.

## Discussion

In this section, we have described few observations about our proposed approaches and these are discussed below:

1. The simulation results favors installing reactive flow rules over proactive flow rules in case of ACL policy change. The proactive mechanism poses a significant risk of underutilizing the Ternary Content Addressable Memory (TCAM) of switches. Moreover, it is challenging to predict the functional lifetime of the flow rule entries that are initially installed, including their frequency (number of entries occurring simultaneously in significant switches of a single domain), velocity (SDN controller message to delete the rule entries urgently), and controller requirements for a deep understanding of all initially installed flow rules. However, the reactive approach is more resilient and identifies problems that must be rectified at the right time. For instance, a policy change requires translation, interpretation of controller duties, and a lengthy transition from the data plane to the control plane. The reactive method only calls the controller functions for flow rule entries generated by the previous policy version. Thus, the findings across several Key Performance Indicators (KPIs) as depicted in the graph, support the effectiveness of the reactive method. The reactive approach to flow rule installation in SDN networks can lead to latency issues, impacting real-time applications and causing delays in network traffic processing. Additionally, the increased load on the controller from handling additional requests for flow rule installation can result in performance degradation and resource exhaustion.
2. The simulation results presented in this study are based on a limited number of ACL policies, hosts, and switches. The proposed solution is designed to be directly used on SDN controllers that support the Pyretic language, offering a flexible and efficient approach for flow rule installation. However, it is also possible to implement the proposed solution on other SDN controllers that utilize a language other than Pyretic. This would require mapping the ACL policies of the alternate language into the format of the ACL policies used in this model, which may require significant effort and careful consideration. Notably, while the proposed solution can be implemented in real-time, we could not conduct a real network implementation due to the unavailability of relevant network traces and a suitable network environment. The results may vary in real-world implementations with many ACL policies, traffic congestion, and a high computation power of the SDN controller.
3. In SDN-based ACL policy implementation scenarios, both reactive and proactive flow rule installation mechanisms may be integrated to harness the benefits of both approaches. The reactive mechanism responds dynamically to network events, allowing for immediate adaptation to changing conditions. This responsiveness enables instant enforcement of ACL policies in response to specific triggers or events, ensuring timely and precise control over network traffic. On the other hand, the proactive flow mechanism involves pre-configuring rules based on anticipated network requirements, thereby preemptively addressing potential issues before they arise. By combining these approaches, SDN controllers can proactively establish baseline policies while also dynamically adjusting them as needed in real-time. This integration may enable the SDN infrastructure to efficiently manage network traffic, optimize resource utilization, and enhance security by swiftly adapting to evolving threats and traffic patterns. However, integrating both these mechanisms presents several challenges. One challenge is ensuring seamless coordination between the two approaches to prevent conflicts and inconsistencies in ACL policy enforcement. Another challenge is managing the complexity of rule sets that result from combining reactive and proactive strategies, as this can lead to increased overhead and potential performance issues. Additionally, reconciling the differing priorities and response times of reactive and proactive mechanisms requires careful design and implementation to achieve optimal results. Moreover, maintaining scalability and flexibility while integrating both approaches can be challenging, especially in dynamic network environments where policies and requirements evolve rapidly. Overall, addressing these challenges requires a comprehensive understanding of the interactions between reactive and proactive mechanisms and careful planning to ensure effective integration while minimizing potential drawbacks.

## Conclusion and future work

This paper presents a comprehensive analysis of proactive and reactive flow rules installation in case of ACL policy change in SDN. The proposed approach in this study incorporates matrices and graph matching techniques to detect changes in ACL policies at different time intervals. The ACL policies are modeled in 5-tuple: Source, Destination, Protocol, Ports, and Action. Once an ACL policy change is detected, the approach calculates new flow rules based on the updated policies. It removes any existing flow rules from switches that conflict with the modified ACL policies. Additionally, it installs new flow rules at switches and stores in the Controller's cache using a hash table data structure, facilitating efficient tracking of flow rules whenever there is an ACL policy change event. Various performance metrics ensure fair experimentation and analysis of the proposed approach. In addition, an educational institute's network topology and ACL policies are used for the simulation. The results demonstrate that reactive flow rules installation yields better results than the proactive mode of flow rules installation in terms of packet violation percentage, successful packet delivery percentage, normalized overhead ratio, network throughput, ACL policy change detection time and average end-to-end delay. This research work can be extended in several ways. The ACL policy change mechanism can be analyzed by incorporating link/node failures to determine the optimal path for flow rule installation. Moreover, the analysis can be expanded to integrate both reactive and proactive flow rule installation mechanisms to maximize the benefits of these approaches for enhancing network efficiency, adaptability, and ACL policy change management. The proposed work can also be implemented in SDN programming languages and controller platforms to assist network administrators in ACL policy implementation. This research work could be integrated with network monitoring and debugging tools to analyze the effectiveness of the policy change mechanism. It may also be investigated how security applications running on SDN controllers and traditional network security protocols behave in more complex scenarios involving ACL policy changes. Lastly, the capabilities of the proposed methodology in managing policy changes can be evaluated in programmable 5G and beyond networks, where guaranteed communication services are highly expected, and there is an increased flux in network reachability rules.

## Data Availability

All data generated or analysed during this study are included in this published article.
