# Author Correction: Efficient handling of ACL policy change in SDN using reactive and proactive flow rule installation

**DOI:** 10.1038/s41598-024-68913-7

**Published:** 2024-08-02

**Authors:** Mudassar Hussain, Rashid Amin, Rahma Gantassi, Asma Hassan Alshehri, Jaroslav Frnda, Syed Mohsan Raza

**Affiliations:** 1 Department of Computer Science and Creative Technologies, Global College of Engineering and Technology, CPO Ruwi 112, P.O Box 2546, Muscat, Sultanate of Oman; 2 Department of Computer Science and IT, University of Chakwal, Chakwal, 48800 Pakistan; 3 Department of Computer Science, University of Engineering and Technology, Taxila, 47050 Pakistan; 4 Department of Electrical Engineering, Chonnam National University, Gwangju, 61186 South Korea; 5 Department of Computer Science, College of Computer Engineering and Science, Prince Sattam Bin Abdulaziz University, Alkharj, 16273 Saudi Arabia; 6 Department of Quantitative Methods and Economic Informatics, Faculty of Operation and Economics of Transport and Communications, University of Zilina, 01026 Zilina, Slovakia; 7 Department of Telecommunications, Faculty of Electrical Engineering and Computer Science, VSB Technical University of Ostrava, 70800 Ostrava, Czech Republic; 8 SAMOVAR, Telecom SudParis, Institut Polytechnique de Paris, 91120 Paris, France

Correction to: *Scientific Reports* 10.1038/s41598-024-65721-x, published online 28 June 2024

The original version of this Article contained errors in the Funding section.

“This research was supported by the Ministry of Education, Youth and Sports of the Czech Republic under the grant SP2023/007 conducted by VSB—Technical University of Ostrava. This research was supported by the Ministry of Education, Youth and Sports of the Czech Republic under the grant SP2023/007 conducted by VSB—Technical University of Ostrava.”

now reads:

“The research was co-funded by the Ministry of Education, Youth and Sports of the Czech Republic (MEYS CZ) within a Student Grant Competition in the VSB – Technical University of Ostrava under project ID No. SGS SP2024/061.”

The original Article has been corrected.

